# Dementia and primary care teams: obstacles to the implementation of Portugal’s Dementia Strategy

**DOI:** 10.1017/S1463423621000876

**Published:** 2022-02-18

**Authors:** Conceição Balsinha, Steve Iliffe, Sónia Dias, Alexandre Freitas, Filipa F. Barreiros, Manuel Gonçalves-Pereira

**Affiliations:** 1 Comprehensive Health Research Centre (CHRC), CEDOC, NOVA Medical School, Faculdade de Ciências Médicas, Universidade Nova de Lisboa, Lisboa, Portugal; 2 Research Department of Primary Care & Population Health, University College London, London, England; 3 Comprehensive Health Research Centre (CHRC), CEDOC, NOVA National School of Public Health, Public Health Research Centre, Universidade NOVA de Lisboa, Lisboa, Portugal; 4 NOVA Medical School, Faculdade de Ciências Médicas, Universidade Nova de Lisboa, Lisboa, Portugal; 5 Logframe, Lisboa, Portugal

**Keywords:** carer, dementia, general practitioner, health services, person with dementia, practice nurse, primary care, qualitative methods, social worker, teamwork

## Abstract

**Background:**

Portugal has a Dementia Strategy that endorses care coordination in the community, but the strategy is not implemented despite there being a network of multidisciplinary primary care clinics that could support it. Recent research into barriers to dementia management in primary care has focused essentially on general practitioners’ (GPs) factors and perspectives. A comprehensive triangulated view on the barriers to dementia management emphasising teamwork is missing.

**Aim:**

To explore the barriers to the implementation of the Portuguese Dementia Strategy by primary care teams, from the perspectives of service users and professionals.

**Methods:**

Purposive sampling was used to recruit 10 GPs, 8 practice nurses, 4 social workers, 8 people with dementia and 10 family carers from 6 practices in different social contexts within the Lisbon metropolitan area. The analytical framework combined codes derived from the transcripts with codes from the available literature. Themes focused on the access to professionals/community services, care coordination within healthcare teams, and between health and community services.

**Findings:**

Several system barriers were identified (undefined roles/coordination within teams, time constraints, insufficient signposting to community services) along with individual barriers (limited competence in dementia, unrecognised autonomy, limited views on social health and quality of life (QoL)), hindering users access to dementia services.

**Conclusion:**

Enhanced competence in dementia, and nurse-led systematic care of people with dementia and their carers, are necessary. They can be effective in improving the QoL in dementia, but only if associated with better community support.

## Background

As the numbers of people with dementia increase, it is unlikely that the current specialist care model will be able to meet their needs. This model is not affordable and does not facilitate continuing care, holistic management of or care coordination for complex multi-morbidities; these are core functions of primary healthcare (Prince *et al.*, 2016).

Currently, there is no cure for dementia, and the goals for clinical care depend on the stage of the disease. Early on, they may focus on maximising function in daily activities and promoting social activities. At least this should be so, according to the recent focus on ‘social health’ in dementia (Dröes *et al.*, 2016). However, in later stages of dementia, the goals may shift to addressing behavioural and psychological symptoms and reducing carer burden. As with other chronic diseases, assessing quality of life (QoL) in dementia is a core task. A metasynthesis of qualitative research identified four relevant determinants (relationships, agency in everyday life, a wellness perspective and a sense of place); the experience of connectedness or disconnectedness within each factor influences the QoL of people with dementia (O’Rourke *et al.*, 2015).

Worldwide, dementia is under-managed in primary care (Prince *et al*., 2016). Three systematic reviews highlight the complex and multifactorial barriers to dementia management (Koch & Iliffe, 2010; Aminzadeh *et al*., 2012; Mansfield *et al*., 2018): people with dementia factors (e.g., non-compliance with care and medication), general practitioner (GP) factors (e.g., lack of knowledge about dementia, unfamiliarity with support services) and system factors (e.g., time constrains; limited availability of support services, and care coordination).

**Figure 1.**
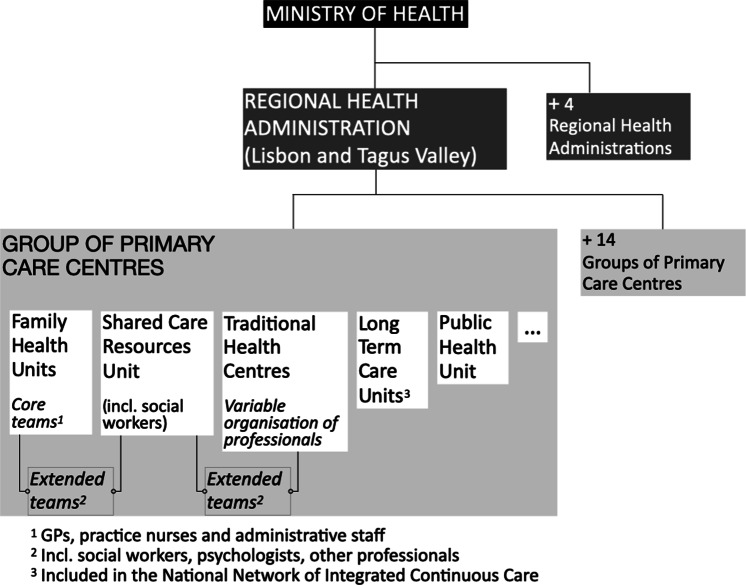
Overview of primary care organisation.

In Portugal, every person has access, in principle, to primary care services within the National Health Service (where GPs control access to secondary care). GPs are considered pivotal health professionals although there is a variable shortage of them, regionally (Gonçalves-Pereira & Leuschner, 2019). In recent years, a new organisation has been introduced in primary care: family health units consisting of multidisciplinary teams (GPs, practice nurses (PNs) and administrative staff) with a variable payment based on capitation and professional performance. The current list of indicators that measure professional performance does not include dementia (Serviços Partilhados Ministério da Saúde, 2021).These units coexist with the traditional health centres, constituting groups of primary care centres, a significant number of them in metropolitan Lisbon (Barros *et al.*, 2011) (Figure 1). The multidisciplinary teams, considered the *core teams*, are supported by an *extended team* (e.g., social workers, psychologists) (Barros *et al.*, 2011).

The area of expertise of the elements of extended teams varies geographically and can comprise social work, psychology, occupational therapy and physiotherapy. However, the ratio between these professionals and the population registered is sometimes very low (e.g., occupational therapists 1:74 126 in one group of primary care centres in Lisbon metropolitan area) (Serviços Partilhados Ministério da Saúde, 2021). Although this new model allows for the structural organisation of the teams, it still falls short of their functional organisation [i.e., team members having explicit functions to achieve a common goal (Bower & Sibbald, 2005)]. In fact, the involvement of PNs in chronic disease management was proposed in 2014 and is still not fully implemented, being in place only for diabetes and hypertension. Other constraints on PNs’ involvement in dementia care are related to insufficient dementia training, and their relative scarcity compared with other EU countries (OECD, 2020). On the whole, concerning dementia management in Portuguese primary care, there are important flaws that should be better characterised. On the whole, concerning dementia management in Portuguese primary care, there are important flaws that should be better characterised. Some concern poor liaison with specialised neurology and psychiatry services and insufficient support to primary care (Balsinha *et al.*, 2019; Gonçalves-Pereira & Leuschner, 2019).

National dementia strategies in Europe emphasise the role of GPs in detecting new cases of dementia and maintaining the general health of the people with dementia; however, their role in diagnosis, initiating anti-dementia drugs and providing social support is more controversial (Koch & Iliffe, 2010). The Portuguese Dementia Strategy which was published only in 2018 (Health Strategy in Dementia, 2018) has yet to be implemented, in part due to the present COVID-19 pandemic constraints. Although indicators were to be defined in regional dementia plans, the strategy outlines different areas of intervention for primary care (Box 1). However, current dementia care pathways in primary care still have a long way to go. Social support for people with dementia is limited, being mostly provided at day care centres that are not specific for dementia, and at home by assistance with basic activities of daily living. Respite services are only available by referral (Balsinha *et al.*, 2019).

In an earlier publications (Balsinha *et al.*, 2021), we explored the role of GPs in dementia care in Portugal and found that GPs contributed little, working alone despite being members of multidisciplinary teams. The provision of dementia care by PNs has been explored in the literature; a recent systematic review identified its potential benefits (e.g., increased patient accessibility to PNs, early recognition and management of cognitive changes, better care management) as well as limitations (e.g., lack of definition of PN roles, inadequate dementia training, time constraints and poor communication with GPs) (Gibson *et al*., 2020).

Despite team-based care being a feature of high-performing primary care (Bodenheimer *et al*., 2014), previous research on the barriers to dementia management in primary care has focused essentially on GPs’ factors (Aminzadeh *et al*., 2012) and perspectives (Koch & Iliffe, 2010; Aminzadeh *et al.*, 2012). In our understanding, a comprehensive triangulated view of barriers to dementia management focusing on teamwork is missing. This could inform future strategies in countries with health systems similar to that of Portugal.

The aim of this study is to explore the obstacles and barriers to the implementation of the Portuguese Dementia Strategy by primary care teams, from the perspectives of service users and professionals.

## Methods

### Design

A qualitative approach was adopted to obtain an in-depth understanding of dementia care (Britten, 2006). Semi-structured face-to-face interviews were conducted. The interview guides drew on available literature (Fortinsky, 2001; Wensing *et al.*, 2009; WHO, 2010, Dreier-Wolfgramm *et al*., 2017) and were adapted for the different sets of participants (see Table 1).

**Table 1.** Interviews’ topic guide

	GPs	PNs	Social workers	Patients	Carers
Introductory questions	x	x	x	x	x
Biographical information	x	x	x	x	x
Access to professionals/community services	x	x	x	x	x
Role in dementia	x	x	x		
QoL determinants in dementia	x	x	x	x	x
Care coordination within healthcare teams	x	x	x		x
Care coordination health-social services	x	x	x		x

GPs – general practitioners; PNs – practice nurses; QoL – quality of life.

Ten pilot interviews (two with each type of participants) were conducted, resulting in minor adaptations to the interview guide (rephrasing a few questions and adding prompts to clarify the answers). Interviews were informal in style, enabling specific issues to be explored as and when they arose (Creswell, 2014).

### Setting

As in our previous studies (Balsinha *et al.,* 2021), four groups of primary care centres within the Lisbon metropolitan area were selected to reflect different socio-economic characteristics.

### Sampling

A contact person (GP) in each family health unit recruited the GPs. Purposive sampling was used to recruit participants (Ritchie *et al*., 2003a). The GPs’ inclusion criterion was that they provided regular care to people with dementia. The GP sample comprised both genders and different durations of clinical experience. PNs belonged to the same core team as the GPs. Social workers were recruited without any specific criteria given their reduced number in each group of primary care centres. People with dementia were recruited by their GPs, if they had a dementia diagnosis according to ICD-10 DCR (WHO, 2004) and could give informed consent. A purposive sample of people with dementia included both genders, individuals at different stages of dementia and with different types of kinship with their carers. All carers were family members. The sample size needed was estimated to be 10–12 participants *per* group, using Guest *et al.*’s methods (Guest *et al.*, 2006).

### Data collection

Data saturation criteria were based on an initial analysis of eight interviews with each group and on a stopping criterion of two interviews where no new ideas would emerge (Francis *et al.*, 2009). These criteria were met at the sixth interview with people with dementia and tenth interview with carers. In the case of GPs and PNs, only in the last interview did new ideas fail to emerge. The criteria were not met in the social workers’ group.

A total of 40 participants were interviewed: 10 GPs, 8 PNs, 4 social workers, 8 people with dementia and 10 carers. Severity of dementia was assessed with the Clinical Dementia Rating (CDR) (Morris, 1993). Two of the 10 people with dementia had advanced dementia and were not able to be interviewed. Three participants had difficulty recalling the care received; therefore, we focused the interview on their subjective experiences (Wilkinson, 2005). The interviews with people with dementia and their carers took place in their homes, and with professionals at their practices. Interviewing was completed before the onset of the COVID-19 pandemic, between March 2018 and May 2019.

Interviews lasted around 40 min and were digitally recorded. Transcriptions were done before the next interview, allowing for new ideas to be explored and discussed by the interviewers (Creswell, 2014). The accuracy of the transcripts was checked by the primary author.

### Data analysis

The framework approach (Ritchie *et al.*, 2003b) and data triangulation were core components of the data analysis. All transcripts were coded by two researchers. Using NVivo 12®, the content of three transcripts was initially examined and the codes generated were grouped into categories. The initial analytical framework drew on these categories and was used to code 15 interviews (3 per group of participants) by 2 of the authors, independently. An analytical framework with six themes was then developed and applied to each transcript, and differences were resolved by discussion.

### Ethics

Ethics approval was granted by the ARSLVT Research Ethics Committee nº 067/CES/INV/2017, and NOVA Medical School Ethics Committee nº 28/2017CEFCM, and written informed consent was obtained from each participant.

### Findings

The characteristics of participants are summarised in Table 2.

**Table 2.** Group characteristics (*n* = 41)

General practitioner (*n* = 10)	
Age, years, median (min-max)	50 (31-64)
Sex, female, *n*	6
Specific postgraduate education in dementia /ageing, *n*	0/0
Years since medical school, median (min-max)	25 (7-41)
Number of people with dementia *per* GP, median (min-max)	12 (5-18)
List size *per* GP, mean (SD)	1850 (98)
Practice nurse (*n* = 7)	
Age, years, median (min-max)	45 (34–58)
Sex, female, *n*	7
Specific postgraduate education in dementia/ageing, *n*	1/1
Years since nursing school, median (min-max)	24 (12-35)
Social worker (*n* = 4)	
Age, years, median (min-max)	47 (38-58)
Sex, female, *n*	4
People with dementia (*n* = 10)	
Age, years, median (min-max)	78 (71-84)
Sex, female, *n*	7
Education, years, median (min-max)	3,5 (0-11)
Living together with carer, *n*	7
Years with dementia since the diagnosis, median (min-max)	3 (1-9)
Dementia type, *n*	
Alzheimer’s disease	6
Vascular dementia	1
Mixed dementia	1
Fronto-temporal dementia	1
Lewy body dementia	1
CDR category 1/2/3, *n*	7/1/2
Having specialist consultations (neurology, psychiatry) for dementia, *n*	8
Receiving support services on behalf of dementia, *n* (CDR category 1/2-3)	5 (3-2)
Time with current GP, years, median (min-max)	10 (1-20)
Carer (*n* = 10)	
Age, years, median (min-max)	61 (44-87)
Sex, female, *n*	7
Education, years, median (min-max)	8 (2-17)
Type of relationship with people with dementia (spouse/child, *n*)	5/5
Dyad has the same GP, *n*	7
Time with current GP, years, median (min-max)	10 (1-20)

**Figure 2.**
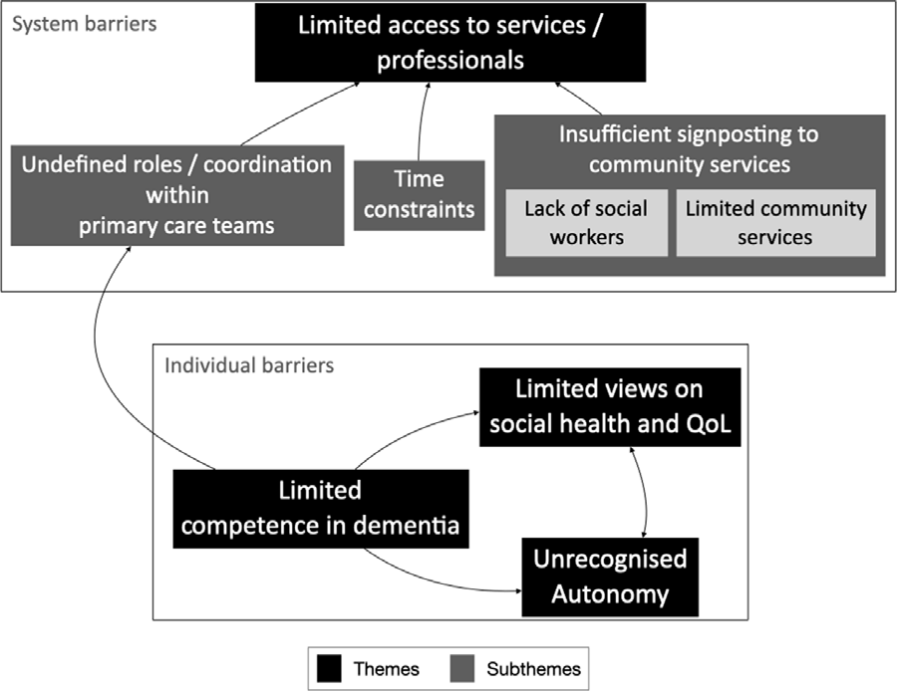
Thematic map.

Four major themes and three subthemes were identified and are summarised in Figure 2.

### Limited access to services/professionals

Most participants considered accessibility to be a fundamental feature in primary care; however, they identified several barriers for patients with dementia and their families to accessing primary care and community services (e.g., home care, day centres).

#### Undefined roles and coordination within primary care teams

All GPs and PNs reported that neither their roles nor coordination within the teams were well defined, which might lead to a dismissive attitude among professionals which could limit access to services, as this GP explained.

We are less involved [in dementia] than in other diseases in which we know our role. It’s difficult to coordinate with the nurse, because it’s not defined as it is for example for diabetes… and altogether this makes us dismissive. (…) Access would be better if there were consultations with nurses. GP5

In fact, most GPs and PNs explained that PNs would only know these patients if they had other chronic conditions.

I’m aware of some patients with dementia, but only those I follow for diabetes or hypertension. PN8

Otherwise, they would provide support for functional dependency only in the advanced stages of dementia.

When they become very dependent, that’s when we visit them. PN7

Only one PN stated that planned coordination within core teams was necessary, similar to other chronic conditions.

We should have definite functions with these patients, as we have with the diabetics. Following them from the beginning of their illness. PN4

Other PNs took a different view; working together with GPs allowed informal coordination on an as-needed basis.

We don’t have a defined role! I’ll go to the GP and say ‘Doctor, I think this lady is becoming forgetful … there’s something wrong with her!’ There is this informality discussing these cases. PN1

None of the social workers questioned their role, but they reported limitations in coordinating with core teams because they were too few to be engaged with each team.

We don’t belong to the core teams (…) it’s difficult to be far from the family health units, but we are very few, so it wouldn’t be possible anyway. SW4

Conversely, all social workers considered the coordination with community services to be good, reporting joint home visits and participation in work groups.

(…) we often do home visits together [with social workers from community services] to assess the situations. SW3

None of the people with dementia referred to professionals other than GPs; some mentioned PNs regarding flu vaccination.

Similarly, carers denied any patient interactions with primary care professionals regarding dementia, other than the GP, with the exception of two carers of patients with advanced dementia whom the nurses assisted regarding functional dependence.

the nurses came here a few times with the GP and told us how we should have our home adapted for this condition (…) C009

#### Time constraints

Some GPs reported having difficulty scheduling patients in general, and most of them reported that consultation time was insufficient. They explained that people with dementia needed assistance with other chronic conditions, their physical examination was more challenging, and that carers presented their own problems.

(…) time is short because these patients are not alone in the consultation and basically there are almost two consultations in one… although often the patient says little, they still have other conditions to be managed(…) GP10

(…) usually adults are able to report their symptoms, but with these patients, we have to pay attention to the physical examination. GP6

A few GPs believed that PNs played an important role in assessing and supporting the carers; however, most nurses denied having time for those tasks. Most often they only provide essential care to people with dementia and carers in intercurrent illnesses and advanced stages. Some PNs regretted this because they felt they should have a role in carers’ psychoeducation.

If we had more time … we’re supposed to teach the families, but we’re always in a rush, we don’t have enough time….PN4

None of the carers reported that the duration of consultations with GPs was insufficient; on the contrary, most of them perceived consultations to be longer than the standard 15–20 min.

The other day the GP was with us for almost an hour. C9

#### Insufficient signposting to community services

Most professionals acknowledged the importance of advising carers about community services. However, two factors contributed to insufficient signposting: lack of social workers and inadequacy or limited availability of those services.

Most GPs felt that signposting to community services was not their function but a task for social workers. Some of them did not know which services were available and did not have enough time to get that information. They usually referred carers to social workers or to PNs when social workers were not available.

When social services are needed, it all becomes complicated. We only have one social worker and that’s not enough. I don’t have time to get to know all the services and… honestly, it’s not my job! It turns out to be the nurse who signposts community services…GP5

I often do the social worker’s job! I briefly signpost the services, and carers do the rest… PN6

We have a social worker one afternoon per week, for a population of 17 000, it’s just not enough. GP7

Moreover, most professionals considered that community services were very few or of limited capacity. They focused on day care centres highlighting their inadequacy for many people with dementia, their strict opening hours, and the lack of a transport service.

There should be day centres adapted for dementia. But there are other problems too… In some places there’s no transportation. If the carer is working till late, who will pick them up at 5 pm? SW2

Some professionals also reported respite care to be insufficient, which might lead to abuse or neglect.

In some cases, these services are urgently needed when there is a risk of neglecting the patient (…) carers are often at their limit, cannot endure it any longer. PN7

None of the carers recalled having met any social worker throughout the referral process. All reported having directly addressed the community services, but some felt lost in the process.

We should’ve been instructed at the family health unit, we had to find the services on our own (…) C8

### Limited competence in dementia

Some professionals highlighted the need to gain competence in dementia care.

Some GPs attributed their own difficulties regarding dementia treatment to the overall limitations of Medicine in this field. Potential for clinical intervention in dementia was underestimated.

It’s hard not to be able to do much for these patients… this is probably one of the great frustrations of medicine in general, right? GP3

For other GPs, having a colleague with a higher level of expertise in dementia could partly overcome the difficulties of speaking directly to specialists.

It would be very important to have someone in family health units who knew a lot about dementia… it’s difficult to reach the hospital … GP2

Some PNs disclosed the negative impact of their lack of competence in dementia on themselves (even their self-esteem) and on the quality of care delivered.

…families often want answers and we sometimes don’t know what to say (…) and we think ‘what could we have done? We didn’t do anything!’… and we feel a little frustrated. PN4

A few people with dementia were unsure regarding their GP’s ability to help them with cognitive decline.

[the GP] seems to help me, but maybe I needed another help for memory problems, another doctor… I don’t know… P7

In line with the professionals, one carer stated that primary care staff needed to be more competent in supporting them.

There should be someone more knowledgeable in how to help families, at the health centre (…) The things I know, came from internet searches…C2

### Unrecognised autonomy

Some GPs acknowledge the impact of the person with dementia’s declining autonomy on care delivery. A few reported being sometimes difficult to have a person-centred attitude in consultations; their uncertainties about patients’ cognitive abilities, associated with a carer’s dominant attitude, might erode the autonomy of people with dementia.

When I see the patient, with the spouse or a child, and it’s difficult to identify the stage of dementia, I end up addressing only the carer … Or when I first address the patient and then the carer immediately contradicts them, I shift my focus to the carer… I feel that the patient is only a bystander and that bothers me …GP10

Conversely, one GP took the view that long-standing relationships with people with dementia and their families made it possible to respect wishes of people with dementia even in advanced stages of disease.

When we accompany these people to the end of their lives, we can work with the families in order to preserve the values and wishes of their relative. GP4

A few professionals and half of the carers attributed some of the people with dementia’s attitudes not to their expression of a will but to mere ‘stubbornness’.

The son worries about her, but she doesn’t want to go to the day centre… she doesn’t want this, she doesn’t want that, she doesn’t want anything! She’s very stubborn! SW1

He’s stubborn, he only does what he wants. If his wife doesn’t stop him, it’s complicated. GP1

The problem is when they don’t want to be helped: when they resist doing the things they should, they’re like children … C5

Conversely, most people with dementia expressed great satisfaction with their ability to make their own decisions. One person explained the importance of having someone who admired their decisiveness.

I have a friend, at the day centre, who likes me a lot … she is everything to me. She thinks I’m determined and that I know what I like and what I want… P5

### Limited views on social health and QoL

The views of most professionals on people with dementia’s QoL were limited to having a family that would keep them safe and support them in their daily activities. Only a few, like GP6, considered it important for people with dementia to have a meaningful occupation and maintain social relationships.

Food and hygiene care must be ensured. (…) Social inclusion is also crucial, as is keeping them occupied with things meaningful to them. GP6

Most of the people with dementia highlighted the importance of social relations but few socialised regularly with friends or neighbours.

(…) and then I have breakfast with a group of friends … knowing that I’m going to meet with them helps me to get out of bed. P7

## Discussion

### Summary

This study describes the experiences of primary care teams and their users regarding barriers to dementia care, in a country without an operationalised Dementia Strategy but where teamwork in primary care should be normal practice. Our findings suggest that the teams lacked a defined role in dementia care, and the users had limited access to dementia services because of several system and individual barriers.

The roles of GPs and PNs were undefined, and their coordination of care for people with dementia was limited, relying on co-location. As a result, some GPs seemed to be unaware of the PNs’ tasks and most participants suggested that GPs were alone in providing care to people with dementia. Surprisingly, most professionals did not seem to attach importance to formal coordination within teams. The lack of social workers and the inadequate community services for people with dementia explain the limited access to those services.

We have also identified individual barriers to dementia care. Some professionals claimed a lack of knowledge about dementia and few relevant skills. Most professionals and carers have a limited view of the QoL and autonomy of people with dementia.

### Strengths and limitations

To the best of our knowledge, this is the first study examining the barriers to dementia management in multidisciplinary primary care teams from the perspectives of team members and service users. The coding and analysis were performed by two authors with experience in consultations with dementia dyads, which could improve their reflexivity. The analytical framework allowed for a combination of inductive and deductive analysis.

There were some limitations. The sample of social workers was limited, but we were able to recruit a social worker in each of the four groups of primary care centres. Our results are not necessarily transferable to other settings; however, primary care teams were drawn from different social settings and at least in Portugal, they could be considered as typical of urban communities and services. Additionally, the Portuguese primary care system is similar to those of other European countries, for example, UK and the Netherlands (Kringos *et al.*, 2015). Purposive sampling may have introduced bias (e.g., people with dementia nominated by their GPs may have better doctor–patient relationships).

### Comparison with existing literature

Our findings suggest that teamwork concerning the needs of people with dementia and their family carers was restricted, which is consistent with previous research (Hinton *et al*., 2007; Mansfield *et al*., 2018). The members of the core teams in our study did not have explicit functions regarding dementia care, a central feature of team-working (Bower & Sibbald, 2005). This was particularly evident in the case of PNs, who mostly delivered opportunistic care, despite previous research suggesting that PNs’ systematic involvement in dementia care can improve assessment, screening, and counselling (Jennings *et al.*, 2016). Previous research suggested that national regulations may affect GPs engagement in dementia care (Petrazzuoli *et al*., 2017). The fact that publication of a Dementia Strategy was late in Portugal, when compared to other European countries, and the current delay in its implementation, can negatively affect the involvement of GPs and PNs in providing specific care in dementia.

This study findings suggest that both GPs and PNs perceive time constraints as a barrier to providing care to their patients with dementia and having a role in carers’ psychoeducation, respectively. Our analysis of the interviews supported and extended previous research that had identified time constraints as a barrier to the management of dementia by GPs (Koch & Iliffe, 2010; Aminzadeh *et al.*, 2012; Mansfield *et al*., 2018).

Our findings also support others showing that GPs do not signpost to community services (Hinton *et al.*, 2007; Pathak & Montgomery, 2015; Foley *et al*., 2017) and are not familiar with them (Pathak & Montgomery, 2015). Despite our finding that PNs would take up the signposting role, they had not received this information from the primary care team. Although it is debatable whether this task belongs to GPs, previous research suggests that people with dementia and their family carers expect to get this information from their family doctor (Foley *et al*., 2017). Finally, the poor signposting of community services may be due to the limited supply of dementia-specific services. Best practice recommendations to improve access to and use of home care services or day care are far from being followed in Portugal, as elsewhere (Stephan *et al*., 2018; Røsvik *et al.*, 2020).

The Interdem consensus on social health and dementia advocates helping people with dementia to manage life despite the disease (Dröes *et al*., 2016); however, our findings suggest that professionals and carers were challenged by the agency of people with dementia. Moreover, most professionals did not recognise the broader QoL and psychosocial needs of their patients, which is also consistent with previous research (Aminzadeh *et al.*, 2012). These negative attitudes may stem from deficits in knowledge, from biased observation of people living with dementia (Gerritsen *et al.*, 2018), and from the need to balance safety versus autonomy (Behrman *et al.*, 2017; Stephan *et al.*, 2018). Importantly, these attitudes contrasted with people with dementia’s appreciation of their agency and relationships, as other studies highlighted (O’Rourke *et al.*, 2015). Primary care services could have an important role in the development of social health in dementia, but this would call for profound attitudinal changes (Gonçalves-Pereira *et al.*, 2021).

### Implications for research, policy and practice

This research has identified obstacles to implementation of Portugal’s Dementia Strategy in primary care. Our results suggest that primary care teams need to extend their functional organisation to dementia care provision, for example, team members having explicit functions to achieve a common goal (Bower & Sibbald, 2005). This could be crucial to improve, for instance, post-diagnostic support (Siva, 2021). Additionally, the extended teams must include a workforce from different disciplines (e.g., social workers, neuropsychologists, psychologists, and occupational therapists) in adequate number to deliver person-centred tailored care to people with dementia and their families.

The lack of community support for people with dementia creates another obstacle to policy implementation. In fact, previous research has highlighted the relevance of psychosocial needs among people with dementia in Portugal (Gonçalves-Pereira *et al*., 2019). Re-designing the primary care teams may not improve *per se* the QoL of many people with dementia and their carers. This would call for community interventions in the form of social work guidance, home care, and re-location to more supportive environments.

To test our hypothesis, an implementation study is needed, with investment in community resources as the primary intervention, and promotion of dementia pathways in primary care being a supplementary intervention.

## References

[ref1] Aminzadeh F , Molnar FJ , Dalziel WB and Ayotte D (2012) A review of barriers and enablers to diagnosis and management of persons with dementia in primary care. Canadian Geriatrics Journal 15, 85–94. 23259021 10.5770/cgj.15.42 PMC3521322

[ref3] Balsinha C , Gonçalves-Pereira M , Iliffe S , Freitas JA and Grave J (2019) Health-care delivery for older people with dementia in primary care. Ch 23. In Lima CADM and Ivbijaro G , editors, Primary care mental health in older people. Switzerland: Springer, 311–329.

[ref4] Balsinha C , Iliffe S , Dias S , Freitas A , Grave J and Gonçalves-Pereira M (2021) What is the present role for general practitioners in dementia care? Experiences of general practitioners, patients and family carers in Portugal. Dementia 20, 1988–2006. 33342279 10.1177/1471301220977710 PMC8358531

[ref5] Barros PP , Machado SR and Simões JA (2011) Portugal – health system review. Health Systems in Transition 13, 1–156. 22222781

[ref6] Behrman S , Wilkinson P , Lloyd H and Vincent C (2017) Patient safety in community dementia services: what can we learn from the experiences of caregivers and healthcare professionals? Age and Ageing 46, 518–521. 27932369 10.1093/ageing/afw220

[ref7] Bodenheimer T , Ghorob A , Willard-Grace R and Grumbach K (2014) The 10 building blocks of high-performing primary care. Annals of Family Medicine 12, 166–171. 24615313 10.1370/afm.1616 PMC3948764

[ref8] Bower P and Sibbald B (2005) The health care team. Ch 1.3. In Jones R , Britten N , Culpepper L , Gass D , Grol R , Mant D and Silagy C , editors, Oxford textbook of primary care. Italy: Oxford University Press, 14–18.

[ref9] Britten N (2006) Qualitative interviews. In Pope C and Mays N , editors, Qualitative research in health care. Oxford: Blackwell Publishing Ltd, 12–20.

[ref10] Creswell J (2014) Research design – quantitative, qualitative and mixed methods approaches. London: SAGE Publications, Inc, 233–261.

[ref11] Dreier-Wolfgramm A , Michalowsky B , Austrom MG , Van Der Marck MA , Iliffe S , Alder C , Vollmar HC , Thyrian JR , Wucherer D , Zwingmann I and Hoffmann W (2017) Dementia care management in primary care: current collaborative care models and the case for interprofessional education. Zeitschrift für Gerontologie und Geriatrie 50, 68–77. 28364258 10.1007/s00391-017-1220-8

[ref12] Dröes RM , Chattat R , Diaz A , Gove D , Graff M , Murphy K , Verbeek H , Vernooij-Dassen M , Clare L , Johannessen A , Roes M , Verhey F and Charras K (2016) Social health and dementia: a European consensus on the operationalization of the concept and directions for research and practice. Aging & Mental Health 21, 4–17. 27869503 10.1080/13607863.2016.1254596

[ref13] Foley T , Boyle S , Jennings A and Smithson WH (2017) “We’re certainly not in our comfort zone”: a qualitative study of GPs’ dementia-care educational needs. BMC Family Practice 18, 66. 28532475 10.1186/s12875-017-0639-8 PMC5441069

[ref14] Fortinsky RH (2001) Health care triads and dementia care: integrative framework and future directions. Aging and Mental Health 5 (Suppl 1), S35–S48. 11513496

[ref15] Francis JJ , Johnston M , Robertson C , Glidewell L , Entwistle V , Eccles MP and Grimshaw JM (2009) What is an adequate sample size? Operationalising data saturation for theory-based interview studies. Psychology & Health 25, 1229–1245. 10.1080/08870440903194015 20204937

[ref16] Gerritsen DL , Oyebode J and Gove D (2018) Ethical implications of the perception and portrayal of dementia. Dementia 17, 596–608. 27288459 10.1177/1471301216654036

[ref17] Gibson C , Goeman D and Pond D (2020) What is the role of the practice nurse in the care of people living with dementia, or cognitive impairment, and their support person(s)? A systematic review. BMC Family Practice 21, 141. 32660419 10.1186/s12875-020-01177-y PMC7359614

[ref18] Gonçalves-Pereira M and Leuschner A (2019) Portugal. In Burns A and Robert P , editors, Dementia care: international perspectives. Oxford: Oxford University Press, 219–230.

[ref20] Gonçalves-Pereira M , Marques MJ and Balsinha C (2021) A demência e as pessoas: importância do conceito ‘saúde social’ e dos cuidados de saúde primários (Persons with dementia: the value of social health and primary health care). Acta Médica Portuguesa 34, 169–170. 33641709 10.20344/amp.15508

[ref44] Gonçalves-Pereira M , Marques MJ , Balsinha C , Fernandes A , Machado AS , Verdelho A , Barahona-Corrêa B , Bárrios H , Guimarães J , Grave J , Alves L , Almeida M C , Reis T A , Orrell M , Woods B , De Vugt M , Verhey F and Actifcare Consortium (2019) Needs for care and service use in dementia: baseline assessment of Portuguese participants in the Actifcare cohort study. Acta Méedica Portuguesa 32, 355–367. 10.20344/amp.11136 31166896

[ref21] Guest G , Bunce A and Johnson L (2006) How many interviews are enough? An experiment with data saturation and variability. Field Methods 18, 59–82.

[ref22] Health Strategy in Dementia (2018) Ministerial Order nº 5988/2018 Republic Diary, 2ª serie, Nº 116 from Office of Assistant Secretary of State and Health. Retrieved 8 February 2021 from https://dre.pt

[ref23] Hinton L , Franz CE , Reddy G , Flores Y , Kravitz RL and Barker JC (2007) Practice constraints, behavioral problems, and dementia care: primary care physicians’ perspectives. Journal of General Internal Medicine 22, 1487–1492. 17823840 10.1007/s11606-007-0317-y PMC2219799

[ref24] Jennings LA , Tan Z , Wenger NS , Cook EA , Han W , Mccreath HE , Serrano KS , Roth CP and Reuben DB (2016) Quality of care provided by a comprehensive dementia care comanagement program. Journal of the American Geriatric Society 64, 1724–1740. 10.1111/jgs.14251 PMC4988879 27355394

[ref26] Koch T and Iliffe S (2010) Rapid appraisal of barriers to the diagnosis and management of patients with dementia in primary care: a systematic review. BMC Family Practice 11, 52. 20594302 10.1186/1471-2296-11-52 PMC2909966

[ref25] Kringos DS , Boerma WGW , Hutchinson A and Saltman R (2015) *Building primary care in a changing Europe: case studies*. Copenhagen: European Observatory on Health Systems and Policies. Retrieved 3 April 2021 from https://www.ncbi.nlm.nih.gov/books/NBK459010/ 29064645

[ref27] Mansfield E , Noble N , Sanson-Fisher R , Mazza D and Bryant J (2018) Primary care physicians’ perceived barriers to optimal dementia care: a systematic review. The Gerontologist 59, e697–e708. 10.1093/geront/gny067 29939234

[ref28] Morris JC (1993) The clinical dementia rating (CDR): current version and scoring rules. Neurology 43, 2412–2414. 10.1212/wnl.43.11.2412-a 8232972

[ref29] O’rourke H , Duggleby W , Fraser K and Jerke L (2015) Factors that affect quality of life from the perspective of people with dementia: a metasynthesis. Journal of the American Geriatric Society 63, 24–38. 10.1111/jgs.13178 25597556

[ref30] OECD (2020) Health at a glance: Europe 2020: state of health in the EU cycle. Paris: OECD Publishing, 218–220.

[ref31] Pathak KP and Montgomery A (2015) General practitioners’ knowledge, practices, and obstacles in the diagnosis and management of dementia. Aging & Mental Health 19, 912–920. 25393131 10.1080/13607863.2014.976170

[ref32] Petrazzuoli F , Vinker S , Koskela TH , Frese T , Buono N , Soler JK and Thulesius H (2017) Exploring dementia management attitudes in primary care: a key informant survey to primary care physicians in 25 European countries. International Psychogeriatrics 29, 1413–1423. 28416036 10.1017/S1041610217000552

[ref33] Prince M , Comas-Herrera MA , Knapp M , Guerchet M and Karagiannidou MM (2016) World Alzheimer report 2016: improving healthcare for people living with dementia – coverage, quality and costs now and in the future. London: Alzheimer’s Disease International, 21–43.

[ref34] Ritchie J , Lewis J and Elam J (2003a) Designing and selecting samples. In Ritchie J and Lewis J , editors, Qualitative research practice. London, Thousand Oaks, CA: Sage, 77–108.

[ref35] Ritchie J , Spencer L and O’connor W (2003b) Carrying out qualitative analysis. In Ritchie J and Lewis J , editors, Qualitative research practice. London: Sage, 219–262.

[ref36] Røsvik J , Michelet M , Engedal K , Bergh S , Bieber A , Gonçalves-Pereira M , Portolani DM , Hopper L , Irving K , Jelley H , Kerpershoek L , Meyer G , Marques MJ , Sjølund BM , Sköldunger A , Stephan A , Verhey F , de Vugt M , Woods B , Wolfs C , Zanetti O and Selbaek G (2020) Development of best practice recommendations to enhance access to and use of formal community care services for people with dementia in Europe: a Delphi process conducted by the Actifcare project. Aging & Mental Health, 25, 2298–2309. 33030026 10.1080/13607863.2020.1822286

[ref37] Serviços Partilhados Ministério da Saúde (2021) Bilhete de Identidade dos Cuidados de Saúde Primários (Primary Care ID). Retrieved 3 April 2021 from https://bicsp.min-saude.pt/pt/Paginas/default.aspx

[ref38] Siva N (2021) New global initiative to tackle Alzheimer’s disease. Lancet (London, England) 397, 568–569. 10.1016/S0140-6736(21)00364-0 33581812

[ref39] Stephan A , Bieber A , Hopper L , Joyce R , Irving K , Zanetti O , Portolani E , Kerpershoek L , Verhey F , de Vugt M , Wolfs C , Eriksen S , Røsvik J , Marques MJ , Gonçalves-Pereira M , Sjölund BM , Jelley H , Woods B , Meyer G and Actifcare Consortium (2018) Barriers and facilitators to the access to and use of formal dementia care: findings of a focus group study with people with dementia, informal carers and health and social care professionals in eight European countries. BMC Geriatrics 18, 131. 29866102 10.1186/s12877-018-0816-1 PMC5987478

[ref40] Wensing M , Mainz J and Grol R (2009) A standardised instrument for patient evaluations of general practice care in Europe. European Journal of General Practice 6, 82–87.

[ref41] WHO (2004) ICD 10: international statistical classification of diseases and related health problems: tenth revision. Geneva: World Health Organization, 45–57.

[ref42] WHO (2010) Primary Care Evaluation Tool, WHO Europe. Retrieved 19 September 2020 from http://www.euro.who.int/__data/assets/pdf_file/0004/107851/PrimaryCareEvalTool.pdf

[ref43] Wilkinson H (2005) Including people with dementia in research: methods and motivations. In Wilkinson H , editors, The perspectives of people with dementia: research methods and motivations. London: Jessica Kingsley Publishers, 10–26.

